# Tumor Necrosis Factor Alpha Deficiency Improves Endothelial Function and Cardiovascular Injury in Deoxycorticosterone Acetate/Salt-Hypertensive Mice

**DOI:** 10.1155/2020/3921074

**Published:** 2020-02-28

**Authors:** Ruiping Cai, Yun Hao, Yue-Yang Liu, Lei Huang, Yang Yao, Ming-Sheng Zhou

**Affiliations:** ^1^Department of Physiology, Shenyang Medical College, Shenyang 110034, China; ^2^Department of Physiology, Jinzhou Medical University, Jinzhou 121001, China; ^3^The Open Project of Key Laboratory of Prevention and Treatment of Cardiovascular and Cerebrovascular Diseases, Ministry of Education, Gannan Medical University, Ganzhou 341000, China

## Abstract

It has been shown that the inflammatory cytokine tumor necrosis factor *α* (TNF*α*) plays a role in the development of hypertension and end-stage renal diseases. We hypothesize that TNF*α* contributes to endothelial dysfunction and cardiac and vascular injury in deoxycorticosterone acetate (DOCA)/salt-hypertensive mice. The wild-type or TNF*α*-deficient mice were uninephrectomized and implanted with DOCA pellet treatment for 5 weeks; the mice were given either tap water or 1% NaCl drinking water. DOCA mice developed hypertension (systolic blood pressure (SBP): 167 ± 5 vs. 110 ± 4 mmHg in control group, *p* < 0.05), cardiac and vascular hypertrophy, and the impairment of endothelium-dependent relaxation to acetylcholine (EDR). TNF*α* deficiency improved EDR and lowered cardiac and vascular hypertrophy with a mild reduction in SBP (152 ± 4 vs. 167 ± 5 mmHg in DOCA group, *p* < 0.05) in DOCA mice. The mRNA expressions of the inflammatory cytokines, including TNF*α*, interleukin 1*β* (IL1*β*), monocyte chemotactic protein 1 (MCP1), and monocyte/macrophage marker F4/80 were significantly increased in the aorta of DOCA-hypertensive mice; TNF*α* deficiency reduced these inflammatory gene expressions. DOCA-hypertensive mice also exhibited an increase in the vascular oxidative fluorescence intensities, the protein expressions of gp91phox and p22phox, and the fibrotic factors transforming growth factor *β* and fibronectin. TNF*α* deficiency reduced oxidative stress and fibrotic protein expressions. The DOCA mice also showed a decrease in the protein expression of eNOS associated with increased miR155 expression; TNF*α* deficiency prevented a decrease in eNOS expression and an increase in miR155 expression in DOCA mice. These results support the idea that TNF*α* significantly contributes to vascular inflammation, vascular dysfunction, and injury in hypertension.

## 1. Introduction

Hypertension is a major risk factor for other cardiovascular diseases, affecting over one billion people worldwide. Uncontrolled hypertension leads to vascular dysfunction and severe end organ damage, such as myocardial infarction, stroke, heart failure, and end-stage renal diseases [1, 2]. Endothelial dysfunction has been recognized as an early marker of abnormalities of vascular function and structure [3]. Increasing evidence suggests that inflammatory cytokines, such as tumor necrosis factor alpha (TNF*α*), play a pivotal role in the induction of vascular dysfunction in cardiovascular and metabolic diseases [4, 5].

TNF*α* is primarily synthesized by the monocytes and macrophages. Other cells, such as lymphocytes, vascular endothelial and smooth muscle cells, fibroblasts, and neuronal cells, can also produce TNF*α* [6]. TNF*α* acts by binding its receptors: TNF receptor type 1 (TNFR1) and type 2 (TNFR2) [6]. These receptors in turn activate multiple signal pathways, including c-Jun N-terminal kinase (JNK), NADPH oxidase activation, and nuclear factor *κ*B (NF*κ*B) [7, 8]. In the endothelial cells, TNF*α* has been shown to inhibit endothelial nitric oxide synthase (eNOS) expression via the destabilization of eNOS mRNA [9] and increase the expression of the adhesion molecules via the activation of the NF*κ*B pathway [10]. In the endothelial and vascular smooth muscle cells, TNF*α* activates NADPH oxidase to induce oxidative stress [11].

Accumulating evidence suggests that TNF*α* plays an important role in the dysregulation of macrovascular and microvascular function in metabolic and inflammatory diseases, such as obesity, diabetic metabolic syndrome, myocardial ischemia/reperfusion, and rheumatoid arthritis [12–14]. It is proposed that hypertension is a chronic vascular inflammatory disease [15, 16]. We have previously shown that salt-sensitive hypertension has severe endothelial dysfunction and end organ damage, which are linked to the activation of the NF*κ*B inflammatory pathway and increased vascular TNF*α* expression [17–19]. Other studies suggest that TNF*α* may participate in the regulation of blood pressure and target organ damage in hypertension [20, 21]. The deoxycorticosterone acetate (DOCA)/salt-hypertensive mouse is a well-established model of salt-sensitive hypertension with severe vascular and renal dysfunction [22]. DOCA/salt hypertension is associated with increased plasma and tissue TNF*α*. Elmarakby et al. [22] have shown that the inhibition of TNF*α* reduces renal injury in the DOCA/salt-hypertensive rats. In the present study, we investigated the role of TNF*α* in endothelial dysfunction and cardiovascular injury using TNF*α*-deficient mice in DOCA/salt hypertension.

## 2. Methods

### 2.1. Animals and Experimental Protocols

Eight-week-old male TNF*α* deficient (TNF*α*^−/−^) mice (the background of C57/BL6 mice) or age-matched wild-type (WT) C57/BL6 mice were purchased from the Model Animal Research Center of Nanjing University (Nanjing, China). All animal protocols comply with the international standards stated in the *Guide for the Care and Use of Laboratory Animals* and are approved by the Institutional Animal Care and Use Committee of Shenyang Medical University. The mice were housed under the conditions of 24°C constant temperature and humidity with a 12 : 12 h light-dark cycle. The mice were adapted to the new environment for two weeks. To induce DOCA/salt hypertension [23], the mice underwent a right nephrectomy via a retroperitoneal incision under ketamine/xylazine anesthesia with 100 mg/kg ketamine/20 mg/kg xylazine (i.p.) cocktail. A 200 mg of 60-day release DOCA pellet (3.3 mg/day/mouse, Innovative Research of American, Sarasota, FL) was implanted in the midscapular region. After recovering from the surgery, the mice were divided into 4 groups and received one of following treatments for 5 weeks: (1) wild-type (WT) control (Ctr, *N* = 8), WT mice with sham surgery and without the implantation of a DOCA pellet; (2) DOCA/salt hypertension (DOCA, *N* = 8), WT mice with a right nephrectomy and DOCA pellet treatment; (3) TNF*α*^−/−^ mice (TNF*α*^−/−^, *N* = 8), TNF*α*^−/−^ mice with sham surgery and without the implantation of a DOCA pellet; (4) TNF*α*^−/−^ with DOCA/salt hypertension (TNF*α*^−/−^/DOCA, *N* = 8), TNF*α*^−/−^ mice with a right nephrectomy and DOCA pellet treatment. The mice receiving the DOCA pellet treatment were also given 1% NaCl and 0.2% KCl to drink for 5 weeks. The control mice underwent sham surgery without DOCA pellet treatment and were fed with tap water. Systolic blood pressure (SBP) was measured in the conscious mice and a quiet environment using the tail-cuff method (Softron Blood Pressure Meter, BP-2010 Series, Tokyo, Japan). The mice were trained daily for 5 consecutive days to adapt for the purpose of blood pressure measurement before the experiments were performed. SBP was measured prior to the implantation of DOCA pellet and once a week during the treatment until the end of the experiment, and at least 5 successive readings of blood pressure were recorded and averaged for each measurement time. At the end of the study, the mice were euthanized by an overdose of anesthetic (sodium pentobarbital 100 mg/kg, i.p.), and the heart and the aorta were harvested.

### 2.2. Histological Analysis

The thoracic descending aorta (just below the highest point of the aortic arch) or a piece of left ventricle (free wall) was fixed in 4% paraformaldehyde in phosphate-buffered saline. The specimens were embedded in paraffin and cut into 4 *μ*m thick sections. The sections were mounted on the slide and then deparaffined. The sections were stained with hematoxylin and eosin (Sigma-Aldrich, St. Louis, MO). For the aorta, four images from four nonconsecutive slides per sample were acquired and analyzed with an Image-Pro Plus version 6.0 software system, and the average aortic wall thickness was measured. The cross-sectional area of 100 cardiomyocytes in 4 randomly selected fields per slide was measured with a quantitative digital image analysis system (Media Cybernetics, Rockville) to assess cardiomyocyte hypertrophy. Masson's trichrome (Sigma-Aldrich, St. Louis, MO) staining was used to evaluate cardiac fibrosis. A semiquantitative analysis of the collagen content in 8 randomly selected fields in two nonconsecutive slides per sample was assessed by evaluating the percentage of positive stained areas with total areas of cardiac tissue with an Image-Pro Plus image analysis system. All histologic samples (8 samples per group) were blind to the reviewers who were not aware of the groups to which the mice belonged.

### 2.3. Organ Chamber Experiments

Acetylcholine-induced endothelium-dependent vasorelaxation in the aortic rings was examined using an organ bath chamber (4-channel Tissue Bath System, DMT Inc. Denmark), as previously described [18]. The aortic rings (the middle part of the thoracic descending aorta) were precontracted to 70% of maximal constriction to norepinephrine (about 30 nmol/L norepinephrine, Sigma-Aldrich, St. Louis, MO); then, an accumulative dose of acetylcholine (10^−9^ to 10^−5^ mol/L, Sigma-Aldrich, St. Louis, MO) was added in the organ chamber. Vascular relaxation to acetylcholine was studied in the intact aortic rings. Maximal response to an agonist (Emax) and the concentration of agonist required for a half-maximal response curve (ED_50_) were determined and calculated from the concentration-response curve, using a best fit to a logistic sigmoid function.

### 2.4. Measurement of Superoxide Anion (O_2_^−^) Production with a Confocal Fluorescence Microscope

O_2_^−^ production in the aortic rings was determined by oxidative fluorescent dye hydroethidine (DHE, Sigma-Aldrich, St. Louis, MO) as previously described [24]. In brief, the fresh aortic rings were embedded in OCT compound; then, the OCT samples were snap-frozen in the liquid nitrogen. The frozen samples were cut into 4 *μ*m thick sections. The slides were submerged in 2 *μ*mol/L dihydroethidine in HEPES buffer and incubated at 37°C for 30 minutes. The images in 8 randomly selected fields in two nonconsecutive slides per sample were acquired by a confocal fluorescence microscope (Leica Microsystems Inc., Mannheim, Germany) within 30 minutes after the incubation with DHE. A double-blind design was used to evaluate dihydroethidium fluorescent intensity for O_2_^−^ production in both endothelium and smooth muscle layers of aortic rings; the average fluorescent intensities were used for the image quantification.

### 2.5. Western Blot

The aortas were homogenized with lysis buffer containing 1 mmol/L PMSF, 10 *μ*g/mL aportinine, and 10 *μ*g/mL leupeptin. Protein concentrations were measured with a BCA Protein Assay Kit (Beyotime Biotech., Shanghai, China). Thirty micrograms of protein was separated by SDS-PAGE and transferred to nitrocellulose membranes (Thermo Fisher, Waltham, MA). Transferred membranes were incubated overnight with specific polyclonal antibodies against gp91phox (Cat#: SC-5827, Santa Cruz Biotech.), p22phox (SC-271262, Santa Cruz Biotech.), transforming growth factor *β* (TGF*β*) (SC-146, Santa Cruz Biotech.), fibronectin (SC-271098, Santa Cruz Biotech.), and eNOS (Cat#: 32027, Cell Signaling). The membranes were incubated with appropriate secondary antibodies (SC-516102, SC-2357, and SC-2354, Santa Cruz Biotech) for 1 hour at room temperature. The signals of luminal chemiluminescent were detected by an Aplegen Omega Lum G Gel Documentation System (Aplegen Inc., Pleasanton) and quantified by ImageJ. The data was normalized to *β*-actin (SC-69879, Santa Cruz) and expressed as fold increase versus the control group.

### 2.6. Real-Time PCR

The aorta was homogenized; total RNA was extracted using the TRIzol reagent (Invitrogen). RNA (2 *μ*g) was reverse-transcribed to cDNA with a superscript II RT first strand synthesis kit (Gibco, BRL) following the manufacturer's instructions. The PCR amplification (real-time PCR instrument, StepOne Plus; ABI) for target genes, including mouse TNF*α*, interleukin *β*1 (IL*β*1), monocyte chemotactic protein 1 (MCP-1), mouse monocyte/macrophage marker F4/80, and endothelial nitric oxide synthases (eNOS), was performed using a PCR amplification kit (TaKaRa Biotechnology Inc. Ltd., Dalian, China). The relative amount of target mRNA was determined using the comparative threshold (Ct) method, and normalized to the values of the housekeeping GADPH. The miR155 cDNAs were synthesized with a Hairpin-it™ miRNAs RT-PCR Quantitation kit (GenePharma, Shanghai, China) following the manufacturer's instruction, and real-time PCR was performed to determine the miR155 levels using U6 snRNA as an internal control. The sequence-specific primers used for the present study were summarized in Table 1. All samples were run in triplicate. Relative quantities of each transcript were normalized by housekeeping gene and expressed as fold increase vs. control.

### 2.7. Data Analysis

All experimental data was expressed as mean ± standard error of the mean (SEM). Statistical analyses were performed using SPSS 18.0 software (SPSS Inc., Chicago, IL), and statistical significance of difference was determined by two-way ANOVA followed with Bonferroni corrections for multiple comparisons between groups. The values were considered significance when *p* < 0.05.

## 3. Results

### 3.1. TNF*α* Knockout (KO) Lowered Blood Pressure, Aortic Hypertrophy, and Cardiac Hypertrophy and Fibrosis in DOCA/Salt Mice

**Figure 1:**
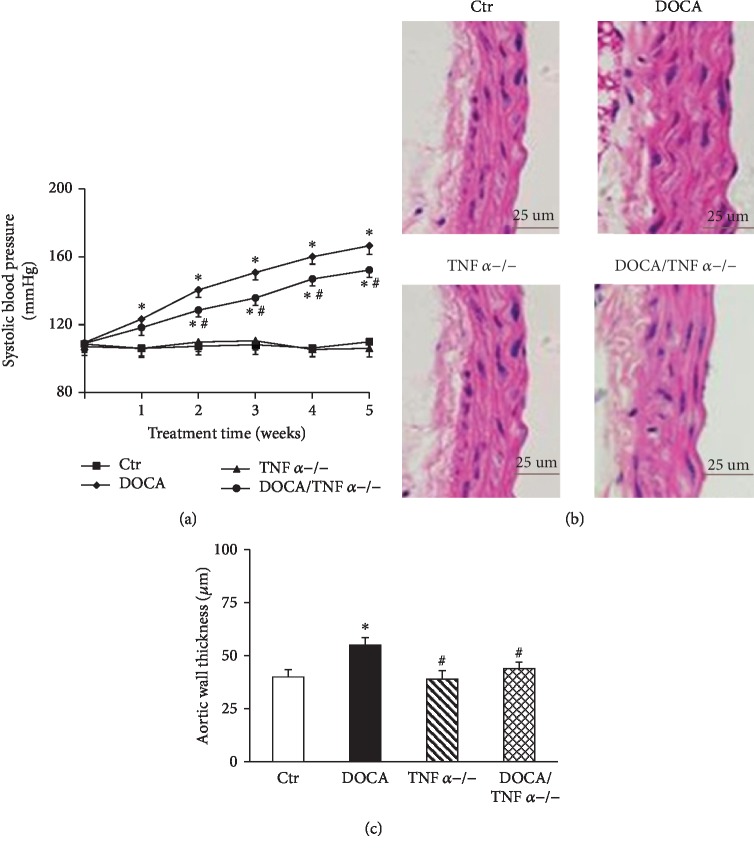
TNF*α* deficiency lowered systolic blood pressure (SBP, *N* = 8) (a) and aortic hypertrophy (b and c) in DOCA/salt-hypertensive mice. (c) The representative images of cross section of the aortic wall stained with hematoxylin and eosin (HE, *N* = 8). (c) The quantitative analysis of aortic wall thickness. Ctr: WT control mice; DOCA: DOCA/salt-hypertensive mice; TNF*α*^−/−^: TNF*α* KO mice; DOCA/TNF*α*^−/−^: TNF*α* KO mice with DOCA/salt treatment. All data were expressed as mean ± SEM. ^∗^*p* < 0.05, vs. the Ctr group; ^#^*p* < 0.05, vs. the DOCA group.

**Figure 2:**
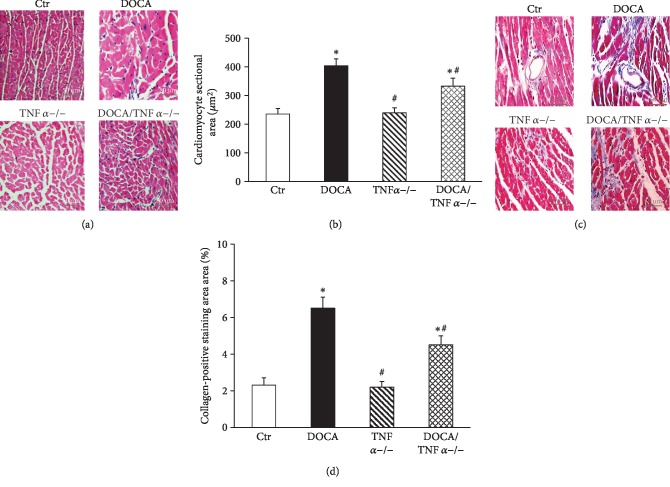
TNF*α* deficiency attenuated cardiac hypertrophy (a and b) and fibrosis (c and d) in DOCA/salt-hypertensive mice. (b) The representative images of cross section of heart stained with hematoxylin and eosin (HE). (b) The quantitative analysis of the cardiomyocyte cross-sectional area (*N* = 8). (c) The representative images of heart section stained with Masson's trichrome for evaluation of cardiac fibrosis. (d) The quantitative assessment of the positive collagen-stained area in the heart (*N* = 8). ^∗^*p* < 0.05, vs. the Ctr group; #*p* < 0.05, vs. the DOCA group.

There was no difference in baseline SBP between WT and TNF*α*-deficient mice. SBP in DOCA/salt mice were significantly increased as compared with the control group (167 ± 5 mmHg vs. 110 ± 4 mmHg in the control group, *p* < 0.05). TNF*α* KO slightly but significantly attenuated the elevation of SBP in DOCA/salt mice (152 ± 4 mmHg vs. 167 ± 5 mmHg in DOCA-hypertensive mice, *p* < 0.05, Figure 1(a)). Neither DOCA/salt treatment nor TNF*α* deficiency affected the heart rate (Table 2). Hematoxylin and eosin staining showed that aortic medial growth and aortic wall thickness significantly increased in DOCA/salt mice compared to the normal control mice. TNF*α* KO significantly reduced the aortic wall thickness in DOCA/salt-hypertensive mice (Figures 1(b) and 1(c)). The cardiomyocyte sectional area also significantly increased in DOCA-hypertensive mice, and TNF*α* KO reduced the cardiomyocyte sectional area in DOCA-hypertensive mice (*p* < 0.05, Figures 2(a) and 2(b). Masson's trichrome staining revealed that DOCA-hypertensive mice had a more positive collagen-stained area when compared with the control mice. TNF*α* KO significantly reduced the positive collagen-stained area in DOCA-hypertensive mice (Figures 2(c) and 2(d)).

### 3.2. TNF*α* KO Reduced Vascular Oxidative Stress in DOCA/Salt-Hypertensive Mice

**Figure 3:**
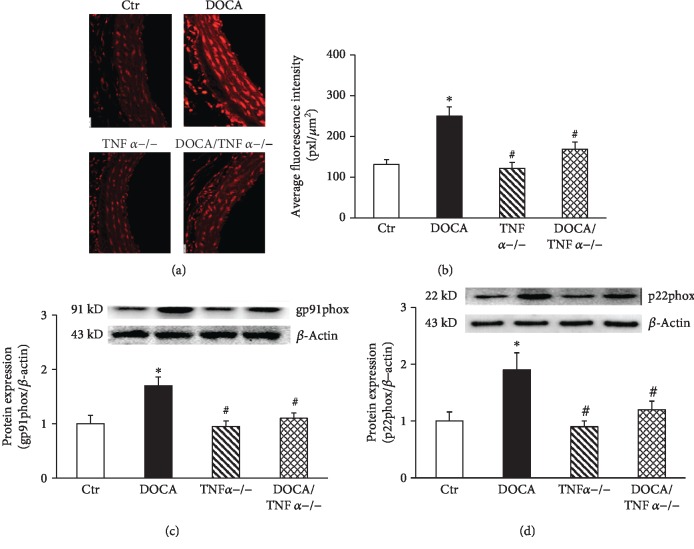
TNF*α* deficiency reduced vascular oxidative stress in DOCA/salt-hypertensive mice. (a) The representative images of aortic oxidative fluorescence intensities stained with DHE assessed by a confocal fluorescence microscope. (b) The quantification of average fluorescence intensities (*N* = 8). (c) The protein expression of NADPH oxidase subunit gp91phox (*N* = 6). (d) The protein expression of NADPH oxidase subunit p22phox (*N* = 6). ^∗^*p* < 0.05, vs. the Ctr group; ^#^*p* < 0.05, vs. the DOCA group.

It has been shown that TNF*α* induces oxidative stress via the activation of NADPH oxidase in endothelium and vascular smooth muscle cells [25]. We used oxidative fluorescent dye dihydroethidine to determine the aortic O_2_^−^ production with a confocal fluorescence microscope. As shown in Figures 3(a) and 3(b), oxidative fluorescence intensities were significantly increased in both endothelium and vascular smooth muscle cells from the aorta of DOCA-hypertensive mice and TNF*α* KO attenuated oxidative fluorescence intensities. The expressions of NADPH oxidase subunits gp91phox and p22phox were significantly increased in the aorta of DOCA-hypertensive mice and reduced in DOCA/TNF*α*^−/−^ mice (Figures 3(b) and 3(c)).

### 3.3. TNF*α* KO Inhibited the Expressions of the Proinflammatory Genes and the Protein Expressions of TGF*β*1 and Fibronectin in the Aorta of DOCA/Salt-Hypertensive Mice

**Figure 4:**
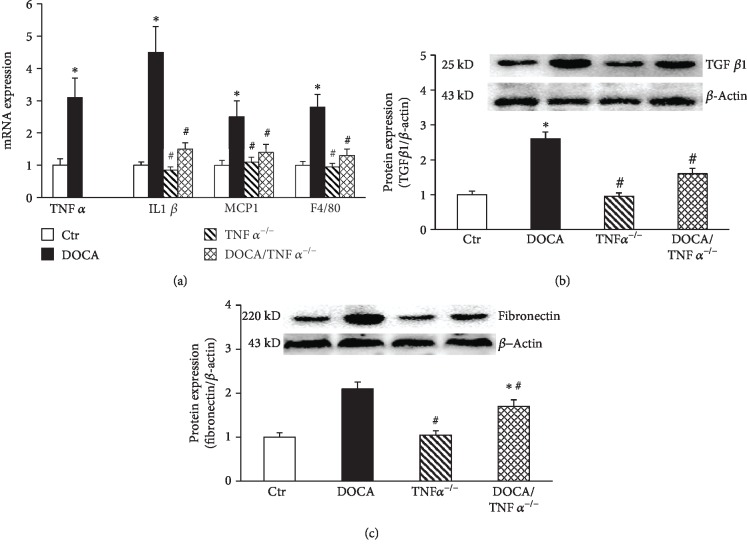
TNF*α* deficiency inhibited the expressions of the proinflammatory genes in the aorta of DOCA/salt-hypertensive mice. (a) The mRNA expression of proinflammatory genes including TNF*α*, IL1*β*, MCP-1, and F4/80 determined by real-time PCR (*N* = 6); the protein expression of fibrotic factors TGF*β*1 (b, *N* = 6) and fibronectin (c, *N* = 6). ^∗^*p* < 0.05, vs. the Ctr group; ^#^*p* < 0.05, vs. the DOCA group.

TNF*α* is one of important proinflammatory cytokines [26]. TNF*α* stimulates the releases of other inflammatory cytokines to induce vascular inflammation and remodeling [26, 27]. As shown in Figure 4(a), the mRNA expressions of proinflammatory cytokines TNF*α*, IL1*β*, MCP-1, and monocyte/macrophage marker F4/80 displayed a 3-5 times increase in the aorta DOCA/salt mice. TNF*α* KO prevented DOCA/salt-induced increases in the mRNA expression of IL1*β*, MCP1, and F4/80. The mRNA expression in TNF*α* KO mice was undetectable (Figure 4(a)). The protein expressions of the fibrotic factors TGF*β* and fibronectin significantly increased in DOCA/salt-hypertensive mice and were found reduced in the DOCA/TNF*α*^−/−^ mice (Figures 4(b) and 4(c)).

### 3.4. TNF*α* KO Increased eNOS Expression and Inhibited miR155 Expression in the Aorta of DOCA/Salt-Hypertensive Mice

**Figure 5:**
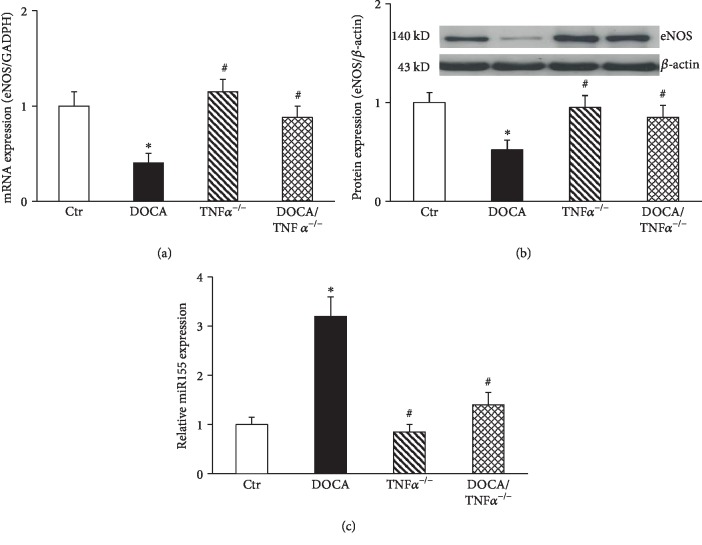
The mRNA (a, *N* = 6) and the protein (b, *N* = 6) expression of eNOS and miR155 expression (c, *N* = 6) in the aorta of DOCA/salt-hypertensive mice. ^∗^*p* < 0.05 vs. the Ctr group; ^#^*p* < 0.05 vs. the DOCA group.

The mRNA and the protein expressions of eNOS were significantly inhibited in DOCA mice. TNF*α* KO prevented reduction in the mRNA and the protein expression of eNOS (Figures 5(a) and 5(b)). It has been shown that TNF*α* inhibits eNOS expression via the induction of miR155 in HUVECs [9]. As shown in Figure 5(c), miR155 expression significantly increased in DOCA/salt mice and was inhibited in DOCA/TNF*α*^−/−^ mice (Figure 5(c)).

### 3.5. TNF*α* KO Improved Endothelial Function in the Aorta of DOCA/Salt-Hypertensive Mice

**Figure 6:**
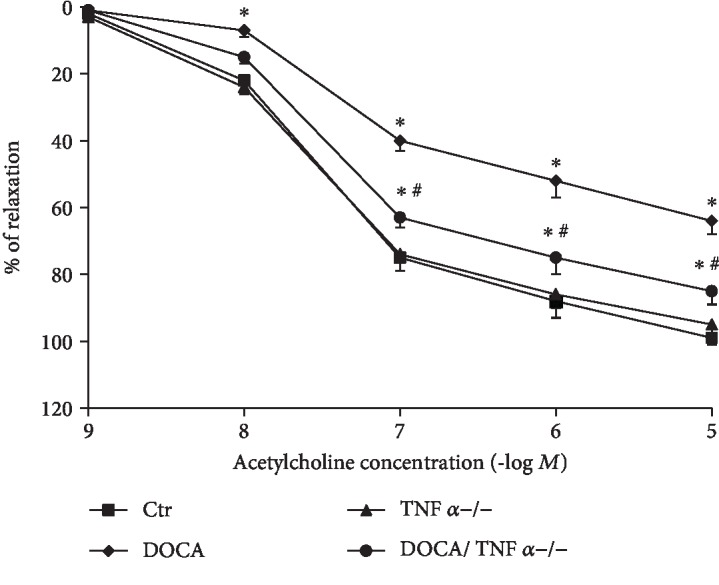
TNF*α* deficiency improved endothelium-dependent relaxation to acetylcholine in DOCA/salt-hypertensive mice. *N* = 6; ^∗^*p* < 0.05, vs. the Ctr group; ^#^*p* < 0.05, vs. the DOCA group.

As shown in Figure 6, endothelium-dependent relaxation to acetylcholine was significantly impaired in DOCA/salt-hypertensive mice (Emax: 65 ± 4 vs. 99 ± 2% in the control group, *p* < 0.05; ED_50_: 7.1 ± 0.15 vs. 7.5 ± 0.12 –log molar in the control group, *p* > 0.05) and TNF*α* KO significantly improved acetylcholine-induced vasorelaxation in DOCA/salt-hypertensive mice (Emax: 85 ± 3 vs. 65 ± 2% in DOCA/salt mice, *p* < 0.05; ED_50_: 7.3 ± 0.2 vs. 7.5 ± 0.1 –log molar in DOCA/salt mice, *p* > 0.05, Table 2).

## 4. Discussion

Accumulating evidence suggests that the immune system and inflammatory cytokines play a critical role in the development of hypertension and target end organ damage [28, 29]. TNF*α* is an important cytokine mainly produced by activated immune cells and is endowed with pleiotropic vascular effects [30, 31]. In the present study, we demonstrated that TNF*α* KO resulted in a blunted response to DOCA/salt-induced elevation of blood pressure, preserved acetylcholine-induced vasorelaxation, and reduced cardiac and vascular injury. TNF*α*-deficient mice also exhibited a reduction in vascular oxidative stress and an increase in eNOS expression associated with the inhibition of vascular miR155 expression.

It has been shown that TNF*α* may act on the vasculature, kidney, and sympathetic nervous system to regulate blood pressure [21, 32, 33]. It has been reported that blockade of TNF*α* with etanercept attenuates either hypertensive response or renal damage in various animal models of hypertension, including preeclampsia, angiotensin II-hypertensive rats, and a model of lupus erythematosus [21, 30, 34, 35]. However, there were also opposite reports that the inhibition of TNF*α* with etanercept failed to reduce blood pressure in Ang II-hypertensive mice and DOCA-salt-hypertensive mice. Although etanercept significantly reduced renal injury [22, 27], the discrepancy on antihypertensive effect of TNF*α* inhibition may be related to animal models or treatment duration. The present study showed that knockout of TNF*α* led to a slight but significant reduction of SBP in DOCA/salt-hypertensive mice. The reduction in SBP started in the 2^nd^ week of DOCA-salt treatment and lasted until the end of the experiment. However, we should explain the result for the small reduction in SBP in TNF*α* KO mice with greater caution because currently there are still some limitations for blood pressure measurement using tail-cuff methods. The inhibition of TNF*α* has been shown to improve renal function and injury in DOCA/salt hypertension [21, 22]; therefore, the improvement of vascular and renal function may be attributed to lowering blood pressure in TNF*α* KO mice.

Recent studies in animal models and humans provide the evidence that show the multiple vascular beneficial effects of anti-TNF*α* therapies in the cardiovascular, metabolic, and inflammatory diseases [14, 31, 36]. In healthy volunteers, intra-arterial TNF*α* infusion could result in acute vascular inflammation associated with impaired endothelium-dependent vasorelaxation [13, 37]. The infusion of TNF*α* worsened endothelial function in type II diabetic mellitus, and the inhibition of TNF*α* was able to improve endothelial function in the patients with rheumatoid arthritis [13]. The target of TNF*α* using either chemical inhibition or genetic KO models has shown to improve endothelial function in the obesity, diabetes, or myocardial ischemia/reperfusion model [12, 38]. However, there were few animal studies to address the role of anti-TNF*α* therapies in vascular dysfunction in hypertension. The present study showed that TNF*α* deficiency improved endothelium-dependent relaxation to acetylcholine in DOCA/salt-hypertensive mice, and the improvement of endothelial function was associated with reduced oxidative stress, vascular inflammation, and the restoration of vascular eNOS expression.

Oxidative stress and inflammation are integral to hypertension-induced vascular and renal injury [28, 39]. We have previously shown that salt-sensitive hypertension is a vascular phenotype which manifests severe loss of endothelium-dependent relaxation associated with oxidative stress and vascular inflammation and the impairment of eNOS-derived NO [17–19]. Either antioxidant treatment or the inhibition of the NF*κ*B inflammatory pathway improved endothelial function and reduced oxidative stress and inflammatory cytokine expression [18]. TNF*α* is an important inflammatory cytokine and potentiates vascular inflammation via the activation of the NF*κ*B inflammatory pathway. TNF*α* can also induce oxidative stress via the stimulation of NADPH oxidase [7] and suppress eNOS expression via increased miRNA155 in HUVECs [9]. Blockade of TNF*α* has proven to have renal protective effects in various animal models of hypertension [22, 35]. Here, we observed that TNF*α* KO in DOCA/salt-hypertensive mice had low levels of oxidative stress and inflammatory gene expression and high eNOS expression. These beneficial vascular effects of TNF*α* KO may be an important contribution to the improvement of endothelial function and reduction of cardiovascular injury in this hypertensive animal model. In addition, we showed that TNF*α* KO suppressed miR155 expression and restored eNOS expression in DOCA/salt-hypertensive mice, and miRNA 155 can inhibit eNOS expression via reduced mRNA stability of eNOS [9]. Therefore, the restoration of eNOS expression in DOCA/TNF*α* KO mice may be related to its inhibition of miRNA155.

In summary, using genetic model of TNF*α* deficiency, we demonstrate that inflammatory cytokine TNF*α* plays a critical role in endothelial dysfunction and cardiac and vascular injury in DOCA/salt hypertension. The inhibition of TNF*α* may have important therapeutic implications for treatment of hypertension and its associated end organ damage.

## Figures and Tables

**Table 1:** PCR primers.

Gene name		Primer sequence (in 5′-3′ direction)
TNF*α*	Forward	CTTCTGTCTACTGAACTTCGGG
	Reverse	CAGGCTTGTCACTCGAATTTTG

IL1*β*	Forward	GGACCCCAAAAGATGAAGGGCTGC
	Reverse	GCTCTTGTTGATGTGCTGCTGCG

MCP-1	Forward	AGGTGTCCCAAAGAAGCTGTA
	Reverse	TCTGGACCCATTCCTTCTTG

F4/80	Forward	CTTTGGCTATGGGCTTCCAGTC
	Reverse	GCAAGGAGGACAGAGTTTATCGTG

eNOS	Forward	CAACGCTACCACGAGGACA
	Reverse	CTCCTGCAAAGAAAAGCTCTG

GADPH	Forward	CTTTGTCAAGCTCATTTCCTGG
	Reverse	TCTTGCTCAGTGTCCTTGC

miR155	Forward	GACTGTTAATGCTAATCGTGATAG
	Reverse	GTGCAGGGTCCGAGGTATTC

U6 SnRNA	Forward	CTCGCTTCGGCAGCACA
	Reverse	AACGCTTCACGAATTTGCGT

TNF*α*: tumor nuclear factor *α*; IL1*β*: interleukin 1*β*; MCP1: monocyte chemotactic protein 1; eNOS: endothelial nitric oxide synthase.

**Table 2:** Heart rate, Emax, and ED_50_.

Variable	Ctr	DOCA	TNF*α*^−/−^	DOCA/TNF*α*^−/−^
Heart rate (beats/min)	534 ± 38	582 ± 45	545 ± 32	562 ± 41
Acetylcholine response				
Emax (%)	99 ± 1	65 ± 4^∗^	95 ± 3^#^	85 ± 4^#^
ED_50_ (−log *M*)	7.5 ± 0.1	7.1 ± 0.2	7.4 ± 0.1	7.3 ± 0.2

^∗^ *p* < 0.05, vs. Ctr; ^#^*p* < 0.05 vs. DOCA. *N* = 8.

## Data Availability

The data used to support the findings of this study are included within the article.
